# Current Measures on Radioactive Contamination in Japan: A Policy Situation Analysis

**DOI:** 10.1371/journal.pone.0152040

**Published:** 2016-03-23

**Authors:** Stuart Gilmour, Shoji Miyagawa, Fumiko Kasuga, Kenji Shibuya

**Affiliations:** 1 Department of Global Health Policy, Graduate School of Medicine, University of Tokyo, Tokyo, Japan; 2 Infectious Diseases Information Surveillance Office, Tuberculosis and Infectious Diseases Control Division, Health Bureau, the Ministry of Health, Labour and Welfare, Tokyo, Japan; 3 Division of Safety Information on Drug, Food and Chemicals, National Institute of Health Sciences, Tokyo, Japan; University of South Carolina, UNITED STATES

## Abstract

**Background:**

The Great East Japan Earthquake on 11^th^ March 2011 and the subsequent Fukushima Dai-ichi nuclear power plant disaster caused radioactive contamination in the surrounding environment. In the immediate aftermath of the accident the Government of Japan placed strict measures on radio-contamination of food, and enhanced radio-contamination monitoring activities. Japan is a pilot country in the WHO Foodborne Disease Burden Epidemiology Reference Group (FERG), and through this initiative has an opportunity to report on policy affecting chemicals and toxins in the food distribution network. Nuclear accidents are extremely rare, and a policy situation analysis of the Japanese government’s response to the Fukushima Dai-ichi nuclear accident is a responsibility of Japanese scientists. This study aims to assess Japan government policies to reduce radio-contamination risk and to identify strategies to strengthen food policies to ensure the best possible response to possible future radiation accidents.

**Methods and Findings:**

We conducted a hand search of all publicly available policy documents issued by the Cabinet Office, the Food Safety Commission, the Ministry of Health, Labor and Welfare (MHLW), the Ministry of Agriculture, Forestry and Fishery (MAFF) and prefectural governments concerning food safety standards and changes to radiation and contamination standards since March 11^th^, 2011. We extracted information on food shipment and sales restrictions, allowable radio-contamination limits, monitoring activities and monitoring results. The standard for allowable radioactive cesium (Cs-134 and Cs-137) of 100 Bq/Kg in general food, 50 Bq/Kg in infant formula and all milk products, and 10 Bq/Kg in drinking water was enforced from April 2012 under the Food Sanitation Law, although a provisional standard on radio-contamination had been applied since the nuclear accident. Restrictions on the commercial sale and distribution of specific meat, vegetable and fish products were released for areas at risk of radioactive contamination. Monitoring of radioactive materials in food products in the prefectures has been mainly conducted before shipment to restrict the distribution of radio-contaminated foods. Between March 2011 and March 2012, 133,832 tests of non-commercial and commercial products were conducted, and 1,204 tests (0.9%) were found to violate the provisional standards. Since April 2012, 278,275 tests were conducted, and 2,372 tests (0.9%) were found to violate the revised standards. MHLW assessment of representative market baskets of foodstuffs at 15 locations throughout Japan between February and March 2014 found very low estimated dietary intake of radioactive cesium (0.0007–0.019 mSv/year), as did assessments of the contents of an average day’s food. Monitoring of fisheries products in coastal areas affected by the nuclear accident found very limited and declining radio-contamination of live fish outside of Fukushima prefecture. Fisheries monitoring is of limited geographical scope and covers only certain fishes.

**Conclusions:**

Area-specific bans on production and distribution have been effective in preventing radioactive contamination in the Japanese food market. Currently there is no major concern about radioactive cesium concentrations in retail foodstuffs in Japan, and very low levels of contamination at the production and wholesale stage. However, because the residue limits and food safety policies were revised on an ad hoc, emergency basis after the nuclear accident, the monitoring procedure needs to be reviewed based on objective and scientifically rational criteria. A transparent and objective scientific framework is needed for prioritizing foodstuffs for inspection and revising Prefecture-specific restrictions. Monitoring of fishes and other seafood products in the wild should be regularized and the information made more publicly accessible, and monitoring activities expanded to identify foodstuffs that are no longer a food safety risk. Consultation with producers and consumers should be more formalized to ensure their concerns are incorporated into regular policy reviews in an appropriate and transparent manner. However, despite the limited available knowledge on best practice in food control and enforcement of provisional radio-contamination limits after the accident, current Japanese policy is sufficient to protect the Japanese public from major risk of radio-contamination from the commercial food market.

## Introduction

On March 11^th^ 2011 Japan’s food safety policies were thrown into turmoil by the Fukushima Dai-ichi nuclear plant accident. This accident occurred in the aftermath of the Great East Japan Earthquake and Tsunami [1], and was rated as a level 7 disaster–the most severe category–by the Nuclear and International Safety Agency (NISA) [2], (which has subsequently been abolished). The accident released radioactive material equivalent to approximately 10% of that released in the Chernobyl accident, leading to the evacuation of nearby communities and the contamination of land downwind of the plant [3, 4]. In an initial assessment of the risk of direct exposure to internal radiocontamination the World Health Organization (WHO) found little risk of increased cancer risk or harmful health effects [5], though both the WHO and an independent investigation established by the National Diet of Japan recommended ongoing monitoring of public health risks [3, 5].

In response to these concerns about radiocontamination through direct exposure to radioactive material before or during evacuation, initial rapid assessments of internal contamination due to Cesium were conducted starting six months after the accident, and reported low risk and levels of exposure [6]. Subsequent studies showed continuing declines in risk of radiation exposure [7, 8], with exposure to potentially contaminated food identified as a risk factor for increased internal contamination[9], especially through consumption of wild-gathered or hunted food products [10]. Levels of internal contamination amongst children living in areas directly affected by the accident are now negligible, and this phenomenon has been associated with effective control of foodstuffs entering the retail marketplace [8, 11].

Nuclear accidents are rare, and there have been only two Level 7 accidents in the history of the nuclear industry. Previous major accidents–Three Mile Island in the USA and Chernobyl in the former Soviet Union–occurred in eras with less sophisticated epidemiological techniques, or in an environment in which independent research could not be conducted. While radioactive material from the Chernobyl accident was distributed widely across Europe [12], assessment of risk factors for internal contamination was not possible for several years after the accident [13], and it was difficult to draw lessons for policy from this accident given the closed nature of the Soviet Union at the time it occurred. Given this limited history of nuclear accidents, the Fukushima dai-ichi nuclear accident presents a unique opportunity to learn lessons for the best food policy response to nuclear disasters, to evaluate the food security measures that were put in place, and to identify improvements in both the current policy and in preparations for the disaster. Given that nuclear power remains an important part of the energy policy mix in both developed and developing nations, it is important to identify robust policy responses to a major nuclear accident, and to inform other nations’ emergency preparedness plans based on Japan’s experience.

In 2011 Japan was selected as a pilot country for the WHO Foodborne Disease Burden Epidemiology Reference Group (FERG) [14]. FERG includes toxins and chemicals in its list of sources of foodborne illness, and in addition to country-specific burden of disease estimation, FERG also includes a framework for the analysis of food safety policy. In this paper we report a policy situation analysis of Japan’s food safety response to the Fukushima dai-ichi nuclear disaster within the framework of the FERG methodology. We will provide a narrative overview of the policy changes that occurred and the emergency measures that were implemented after the disaster, assess the effectiveness of the policy on the basis of available data, and evaluate the policy framework.

## Methods

FERG policy situation analysis guidelines stipulate that the policy process be assessed with respect to the way in which national policy agendas are set, formulated, adopted and evaluated [15]. Consistent with this methodology, we conducted a hand search of all publicly available documents regarding food safety policy changes, evaluation and monitoring from the Cabinet Office, the Food Safety Commission, the Ministry of Health, Labor and Welfare (MHLW), the Ministry of Agriculture, Forestry and Fishery (MAFF) and prefectural governments. This hand search was conducted on all documents published between March 11, 2011 and December 31^st^, 2014. Where documents referred to legislation set before March 11, 2011 this legislation was also searched as necessary. The documents searched included information pamphlets, reports on monitoring processes and outcomes, and descriptions of the food safety legislative framework and responsible organizations. We extracted information from these documents on the timing of changes to policy on allowable sale of foodstuffs, restrictions on commercial production and distribution, and evaluation and monitoring of foodstuffs at the wholesale or retail level. We also extracted information on the results of monitoring tests. These documents were all published in Japanese. We did not incorporate any information on monitoring or evaluation conducted by independent groups, academics or private food assessment companies, as the focus of this analysis was the government’s response and food safety monitoring practices. Our analysis did not include a systematic review of published literature on food safety, as this is not part of the current FERG policy situation analysis framework.

Consistent with FERG policy situation analysis guidelines [15], in this study we assessed the policy situation from the perspective of four major stakeholders with potentially conflicting views: the public; food producers and retailers, whose interests may conflict; and the government agencies responsible for food security, who were required to balance the interests of public health and commercial food production in an environment of rapidly changing information and deep public concern about the health effects of radiation.

Results are presented in terms of the timeline of changes enacted, Prefecture-specific limitations and restrictions, and location and timing of monitoring practices. Food safety measures for preventing internal contamination primarily focus on the risk of Cesium 134 and Cesium 137 radionuclides, as the half-life of Iodine is such that it will no longer be detectable by the time of establishment of enhanced monitoring, and other less volatile radionuclides (such as Strontium) are not expected to form a major component of the internal contamination risk profile after this accident [16], and have not been identified in large quantities in the environment [17]. Residue limits are reported as concentrations (Bq/Kg), and annual committed doses in mSv/year, as they are reported in the documents.

## Results

In March 2011, immediately after the accident, the government of Japan issued provisional guidance on food safety containing revised food residue limits of Cesium [18]. This *Urgent Notice on Radioactive Materials* also limited the sale and distribution of specific products from Fukushima. From April 2012, the *Food Safety Basic Act* was revised to formalize residue limits and consumption restrictions. Monitoring and evaluation tests were also conducted over this period. Table 1 summarizes the residue limits before and after the revision of the *Food Safety Basic Act* and the time periods in which they applied.

**Table 1:** Residue limits in Japanese food after the Fukushima dai ichi nuclear accident, by time period.

Food type	Time period	
	March 17, 2011 –March 21^st^ 2012	April 1 2012 onward
General foods	500 Bq/Kg	100 Bq/Kg
Milk	200 Bq/Kg	50 Bq/Kg
Water	200 Bq/Kg	10 Bq/Kg
Infant foods	200 Bq/Kg	50 Bq/Kg

The March 2011 provisional residue limits were set based on guidelines from the Nuclear Safety Commission. These guidelines were then reviewed by the Food Safety Commission of Japan, and a set of permanent guidelines developed for adoption in the *Food Safety Basic Ac*t. These revised guidelines were intended to ensure that ordinary human consumption of regulated food could not lead to a total annual radiation exposure of more than 1mSv, and were developed based on assignment of contamination fractions by causal pathway [19]. Because little is known about the vulnerability of infants to internal contamination through food, a further set of restrictions on contents of infant formula were introduced. Food monitoring implemented in March 2011 tested a wide range of foodstuffs against these residue limits. In the first year of testing 133,832 tests were conducted of which 1,204 (0.9%) were over allowable residue limits, and a further 278,275 tests were conducted in the second year, with 2,372 (0.9%) also over the allowable residue limits. Table 2 summarizes the results of those tests that were over the residue limits in each year.

**Table 2:** Tests over residue limits by Prefecture and food type, 2011–2013.

Year, product	Non-commercial		Commercial	
	Number of tests	Number detected (%)	Number of tests	Number detected (%)
March 18, 2011 –March 31, 2012				
Milk and dairy	1316	14 (1.1)	1631	9 (0.6)
Vegetables^1^	9810	238 (2.4)	11181	212 (1.9)
Grain	4215	1 (0.0)	1326	1 (0.1)
Meat and eggs^2^	85419	148 (0.2)	5997	137 (2.3)
Fishery products	5485	135 (2.5)	3700	112 (3.0)
Other	2209	144 (6.5)	1543	53 (3.4)
April 1, 2012 –March 31, 2013				
Drinking water	797	13 (1.6)	876	0 (0.0)
Milk and infant food	2522	0 (0.0)	2692	0 (0.0)
Agricultural products^1^	39636	636 (1.6)	10010	5 (0.0)
Livestock products	187883	4 (0.0)	1441	0 (0.0)
Fishery products	17435	1070 (6.1)	3654	2 (0.1)
Wild animal meats	1246	492 (39.0)	8	1 (12.5)
Other	2516	138 (5.5)	7559	11 (0.1)
April 1, 2013 –March 31, 2014				
Drinking water	397	0 (0.0)	741	0 (0.0)
Milk and infant food	2179	0 (0.0)	2805	0 (0.0)
Agricultural products^1^	34425	271 (0.8)	12083	7 (0.1)
Livestock products	246140	0 (0.0)	1690	0 (0.0)
Fishery products	19739	301 (1.5)	3173	0 (0.0)
Wild animal meats	1405	417 (30)	9	0 (0.0)
Other	2610	29 (1.1)	8485	(0.0)

1. Includes wild mushrooms and wild vegetables

2. Includes wild meats

**Fig 1:**
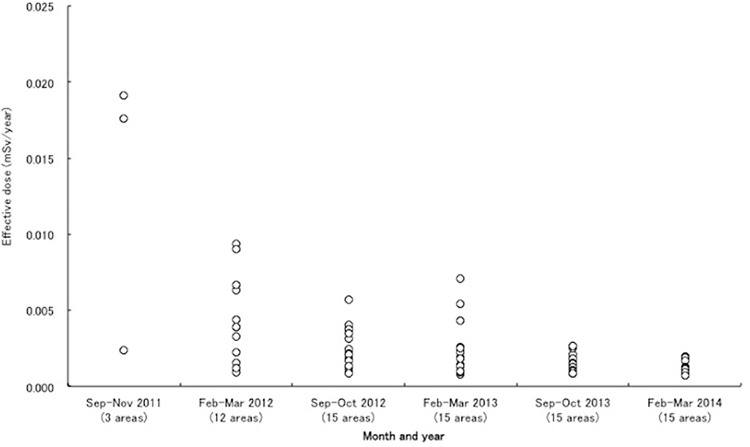
Committed effective doses of Cs134 and Cs137 estimated from market basket samples, 2011–2014.

The MHLW also administered a program of testing market baskets of fresh produce, commencing in September 2011 and conducted biannually over a two month period in a number of locations across Japan. These assessments found an estimated committed effective dose of Cesium for a standard Japanese diet would lie between 0.0007 and 0.019 milliSievert (mSv/year), well below the contamination threshold of 1 mSv/year set by the MHLW. Fig 1 shows these estimated committed effective doses by year and testing period, showing a clear decline in contamination over time.

Since March 2011 the MHLW also implemented monitoring of a common fish widely distributed in Japanese water, the ocellate spot skate (*Okamejei kenojei*) as a potential sentinel species. This research program, which is largely still in its infancy, enabled the comparison of contamination of this common fish between marine areas affected by the nuclear accident and marine areas that are likely uncontaminated. Table 3 summarizes the findings of this program, which was conducted at coastal sites in five Prefectures, three of which (Fukushima, Ibaraki and Miyagi) were affected by the nuclear accident. Internal contamination tests on these skates had a minimum detectable contamination threshold of 20 Bq/Kg, and Table 3 shows the proportion of samples above this detectable threshold, the maximum observed contamination density and the interquartile range of density. Table 3 shows that the proportion of contaminated skate declined over time in the three affected prefectures, and while there was no decline in maximum concentrations the interquartile range narrowed, so that observed maxima became less and less likely to occur over time. There was no evidence of spread of contamination beyond the three affected prefectures.

**Table 3:** Results of assessment of radiocontamination of Ocellate spot skate in five Prefectures, 2011–2013.

Prefecture and year	Number of samples	Number over residue threshold	Percent over threshold	Maximum contamination (Bq/Kg)	Interquartile range (Bq/Kg)
2011					
Fukushima	150	149	99.3	840	59–305
Ibaraki	12	8	66.7	100	24–96
Miyagi	2	0	0.0		
Iwate	1	0	0.0		
2012					
Fukushima	165	161	97.8	850	71–255
Ibaraki	74	50	67.6	110	24–64
Miyagi	8	4	50.0	57	28–55
Iwate	2	0	0.0		
2013					
Fukushima	184	157	85.3	320	40–93
Ibaraki	70	27	38.6	520	25–55
Miyagi	17	0	0.0		
Iwate	8	0	0.0		
Chiba	5	0	0.0		

These results suggest that marine contamination is a concern only in the immediate vicinity of the nuclear powerplant, and that levels of contamination of fishes have been declining over time. Nonetheless, the levels of contamination in fish caught in Fukushima presented a cause for concern, and the small number of samples of single sentinel fish species from other waters are insufficient to draw any firm conclusions about the degree of marine contamination.

Given what is known about the risk of contamination of wild-sourced foods (such as fish, wild fowl and wild boar) and the heightened risk to certain domestic crops such as mushrooms and beef, the MHLW introduced Prefecture-specific restrictions on the distribution and sale of certain foodstuffs, which essentially outlawed the commercial sale, distribution or use of these products, though it did not place any restrictions on personal consumption. These restrictions have evolved steadily over time, and the restrictions as at December 2013 are summarized in Table 4.

**Table 4:** Restrictions on goods for sale or distribution by Prefecture, December 2013.

Prefecture	Products
Fukushima (selected parts)	Whole milk; spinach and other green leafy vegetables; cabbage and broccoli varietals; turnips; log-grown *shiitake* and *nameko* mushrooms; all wild mushrooms; fatsia sprouts; paddock-grown wasabi; fern shoots; wild mountain vegetables; *yuzu* citrus fruits; chestnuts; kiwi fruit; soya and azuki beans; rice grown between 2011 and 2013; Japanese dace; char; eel; sweetfish and carp (except cultured); bear meat
Fukushima (entire prefecture)	Beef, boar, duck, pheasant and hare meat; 40 kinds of fishery products
Aomori (selected parts)	Wild-grown mushrooms
Iwate (selected parts)	Log-grown *kuritake*, *nameko* and *shiitake* mushrooms; all forms of wild mushrooms; bamboo shoots; Japanese fern, Japanese parsley, *koshiabura* flowers, bracken; soya beans; mackerel, perch, bream, char and Japanese dace (except cultured)
Iwate (all parts)	Beef, deer, bear and pheasant meat
Miyagi (selected parts)	Log-grown *shiitake* mushrooms; all forms of wild mushroom; bamboo shoots; fern fronds and *koshiabura* flowers; rice grown in 2013; soya beans; mackerel; puffer fish; trout, char and sweetfish (except cultured); Japanese dace
Yamagata (all parts)	Beef and bear meat
Ibaraki (selected parts)	Log-grown *shiitake* mushrooms; bamboo shoots; *koshiabura* flowers; sole and flounder; American catfish and and crucian carp (except cultured); eel
Ibaraki (all parts)	Boar meat; ocellate spotted skate, Japanese rockfish, perch, croaker, Pacific cod
Tochigi (selected parts)	Log-grown *shiitake*, *nameko* and *kuritake* mushrooms; all forms of wild mushroom; bamboo and fern shoots; *koshiabura* flowers; wild-grown Japanese pepper; fatsia sprouts; bracken; chestnuts
Tochigi (all parts)	Beef, boar and deer meat
Gunma (selected parts)	All forms of wild mushroom; char and trout (except cultured)
Gunma (all parts)	Boar, bear, deer and pheasant meat
Saitama (selected parts)	All forms of wild mushroom
Chiba (selected parts)	Log-grown *shiitake* mushrooms; bamboo shoots; koi and crucia carp; eel
Chiba (all parts)	Boar meat
Niigata (selected parts)	Bear meat
Yamanashi (selected parts)	All forms of wild mushroom
Nagano (selected parts)	All forms of wild mushroom
Shizuoka (selected parts)	All forms of wild mushroom

Source: Ministry of Health, Labor and Welfare, Pharmaceutical and Food Safety Bureau, Department of Food Safety[20]

Due to the urgent nature of initial responses to the nuclear accident, provisional guidelines established for food residue limits on 17^th^ March, 2011 were implemented without public consultation. However, the Food Safety Commission has a standard mechanism for revising the *Food Safety Basic Act* that is intended to ensure transparency and the right of citizens to comment [21]. Consistent with this basic process, a formal process of consultation was conducted in 2011 under the measures required by the *Food Safety Basic Act*, and the laws governing allowable residue limits were formally modified to incorporate the information known about radiation exposure risk, results of monitoring and input from stakeholders and scientists under this process.

**Fig 2:**
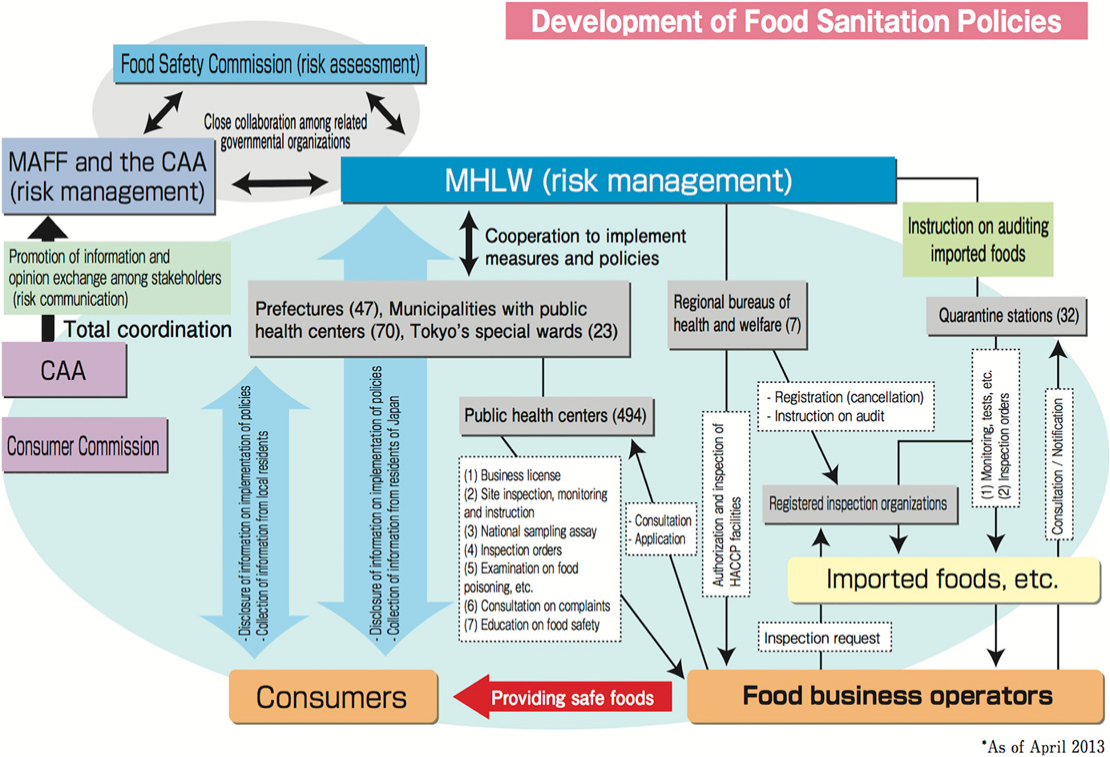
Responsibilities and roles of different stakeholders in development and implementation of food safety policy.

Having established these residue limits, food monitoring and risk communication procedures could be conducted as part of the routine administration of the *Food Safety Basic Act* [21]. This monitoring process involves cooperation between local, Prefectural and national government, and inspection of foods by public health centers and regional bureaus of health welfare. Fig 2 shows the mechanism by which the risk management, risk communication and food safety policies are developed in Japan, and the agencies responsible.

Although initial response to the nuclear accident allowed only the implementation of provisional guidelines, the existing mechanisms of the *Food Safety Basic Act* allowed for a short period of review and public consultation before the development of a permanent set of standards for the management of food risks after the nuclear accident. Consultation with consumers and producers take the form of informal dialogues and public meetings during the development of the policy [22], making assessment of the quality and content of the consultation process difficult.

## Discussion

Radiation contamination countermeasures changed significantly in Japan in the two years since the Great East Japan Earthquake and Tsunami, in the context of a rapidly-evolving major radiation disaster. Initially the government introduced a rapid reaction, provisional policy that was reformed a year after the disaster in the context of a coherent policy revision process. In this study we have applied some components of the FERG policy situation analysis framework to assess the policy changes that occurred at this time, and to review the process of change. We accessed all publicly available documents in order to obtain a comprehensive understanding of the policy context, and obtained publicly available information on the results of monitoring and evaluation studies conducted in the regions affected by the nuclear accident.

This is the first and only analysis of policy changes occurring in the aftermath of a major nuclear accident. Although similarities between the Three Mile Island disaster and the Fukushima accident have been identified [23], there have been no significant analyses of foodborne disease policy changes in the aftermath of that accident, and the initial response to the Chernobyl accident was not studied. Japan’s experience of rapid food safety policy changes after the initial release of radionuclides therefore offers a unique opportunity to assess the effectiveness of food safety countermeasures in preventing foodborne illness due to radiation accidents, and to provide lessons for other countries with a nuclear energy industry.

We found that the Japanese government responded rapidly to the nuclear accident through provisional changes to the law, to set strict thresholds on allowable radiation residues in food and to implement a monitoring framework. While the government of the time was criticized for its response to the release of airborne radionuclides [3], did not enact an iodine exposure screening program immediately after the accident and had to rely on subsequent Prefecture-level screening of thyroid abnormalities to identify those potentially at risk of cancer [24], its policy response to the risks to the human food chain was rapid and comprehensive. We found that, although there is evidence of contamination of seafood, monitoring of commercial food supplies through random testing found a very low risk of any radiocontaminated food entering the food supply, and no evidence of significant risk of internal contamination through consumption of food purchased at retailers. Furthermore, the low risk identified in this study may well be an over-estimate of the true population risk, since the sample testing reported here may have been conducted in a semi-purposive manner, targeting higher-risk areas or products in the aftermath of the accident. Our findings are consistent with independent research studies of radiation exposure in Minamisoma [7, 9, 25], Kawauchi [26] and Iwaki [27] within six months to two years after the accident, and confirms the importance of rapid, early intervention in wholesale food markets and distribution processes. Our review suggests that initial, rapid response through provisional restrictions followed by a more comprehensive policy review process as part of an established food safety framework (in this case, the *Food Safety Basic Act*) was sufficient to protect the public from major radiation contamination threats.

Japan’s initial reaction to the nuclear accident, sustained until the present, included Prefecture-specific bans on the commercial production and distribution of specific foods. Investigations of ocellate spot skate suggest that these bans may have been important in preventing consumption of contaminated fish caught in Fukushima prefecture, but the results from neighbouring Prefectures suggest that these prefecture-specific bans may have been overly restrictive, and may have led to unnecessary restrictions on food producers from other prefectures. The low proportion of foods tested above allowable thresholds in random, systematic monitoring suggests that prefecture-specific food distribution restrictions are effective, but may have caused unnecessary disruption for producers and retailers, and the absence of detailed research or supporting data from the policy documents makes it impossible to evaluate the sensitivity of this policy intervention. Furthermore, the levels of contamination identified in retail products in this study, although above the allowable limits in Japan, were generally below those of other countries, suggesting that some disruption to retailers and producers could have been avoided by limits more in keeping with international standards. Current limits, set at 100 Bq/Kg, are well below those set for imported foods in the USA (1200 Bq/Kg)[28] for example, and none of the identified foods that were above allowable limits in Japan would have been considered unacceptable in the USA. Given the low level of identified risk and the potential damage done to producers, retailers and distributors by their continued application, it may be time for the Japanese government to consider a new risk assessment of radioactive contamination in food.

While monitoring of foodstuffs entering the market from producers and distributors has been extensive and consistent, monitoring and evaluation activities in support of a broader understanding of the policy framework are still needed. For example, detailed data on radioactive contamination by fish type and location are not available for monitoring of fish beyond the ocellate spot skate, and there are no details of any comprehensive monitoring of other foods subject to prefecture-specific production and distribution restrictions. This makes it difficult to assess the scientific basis on which the prefecture-specific restrictions are implemented or reviewed, and raises the possibility that producers from some prefectures are being locked out of food markets without a strong scientific basis.

While the food safety policy has been effective in preventing contamination of commercially available food, risk communication measures could be improved. In the absence of research collaborations with producers and fisheries to obtain estimates of the radiation contamination risk for specific products, it is not possible to remove foods from the restricted list on a coherent basis. Furthermore, some products that are known from previous nuclear contamination incidents to have a higher risk of radiation contamination[12], such as wild vegetables, mushrooms and meats[29], were not separately documented in publicly available reports, making assessment of the separate, higher risk due to these products impossible. A more consistent and transparent review process would serve to ease public concern about the responsiveness of food safety policy-makers, and better public availability of data and monitoring results would offer producers and consumers more input into the consultation process.

## Conclusion

Our policy situation analysis found that the Japanese government response to the risk of radiation contamination of the human food chain was rapid, comprehensive and effective. We found no evidence of serious risk to human health from radiation either in the immediate aftermath of the disaster or in the long term follow up, even though initial policy changes had to be made quickly and with only minimal amounts of preparation. While the food monitoring process is extensive and systematic, better and more detailed data and reports to producers and consumers would help to ensure that foods are not unnecessarily restricted and that the needs of public safety are properly balanced against the needs of local food producers and distributors, and a more formalized consultation and assessment process should be considered to improve dialogue between food safety authorities and the public. However, the Japanese response to the nuclear accident has, to the best of our knowledge, effectively protected the community from serious risk of radiation contamination, and other countries with a nuclear industry should consider adopting the lessons of the Japanese experience as soon as possible, to ensure that they are prepared for the worst consequences of a nuclear accident, and able to protect the health of the public as effectively as Japan has done.
